# Deep Brain Stimulation and Drug-Resistant Epilepsy: A Review of the Literature

**DOI:** 10.3389/fneur.2019.00601

**Published:** 2019-06-06

**Authors:** Nasser Zangiabadi, Lady Diana Ladino, Farzad Sina, Juan Pablo Orozco-Hernández, Alexandra Carter, José Francisco Téllez-Zenteno

**Affiliations:** ^1^Shefa Neuroscience Research Center, Khatam Alanbia Hospital, Tehran, Iran; ^2^Neuroscience Research Center, Institute of Neuropharmacology, Kerman University of Medical Sciences, Kerman, Iran; ^3^Epilepsy Program, Hospital Pablo Tobón Uribe, Neuroclinica, University of Antioquia, Medellín, Colombia; ^4^Department of Neurology, Rasool Akram Hospital, IUMS, Tehran, Iran; ^5^Departamento de Investigación Clínica, Facultad de Ciencias de la Salud, Universidad Tecnológica de Pereira-Clínica Comfamiliar, Pereira, Colombia; ^6^Saskatchewan Epilepsy Program, Department of Medicine, University of Saskatchewan, Saskatoon, SK, Canada

**Keywords:** anterior thalamic nucleus, electrical stimulation, neuromodulation, neurostimulation, refractory epilepsy, target

## Abstract

**Introduction:** Deep brain stimulation is a safe and effective neurointerventional technique for the treatment of movement disorders. Electrical stimulation of subcortical structures may exert a control on seizure generators initiating epileptic activities. The aim of this review is to present the targets of the deep brain stimulation for the treatment of drug-resistant epilepsy.

**Methods:** We performed a structured review of the literature from 1980 to 2018 using Medline and PubMed. Articles assessing the impact of deep brain stimulation on seizure frequency in patients with DRE were selected. Meta-analyses, randomized controlled trials, and observational studies were included.

**Results:** To date, deep brain stimulation of various neural targets has been investigated in animal experiments and humans. This article presents the use of stimulation of the anterior and centromedian nucleus of the thalamus, hippocampus, basal ganglia, cerebellum and hypothalamus. Anterior thalamic stimulation has demonstrated efficacy and there is evidence to recommend it as the target of choice.

**Conclusion:** Deep brain stimulation for seizures may be an option in patients with drug-resistant epilepsy. Anterior thalamic nucleus stimulation could be recommended over other targets.

## Introduction

Deep brain stimulation (DBS) is a neurointerventional technique that involves implanting electrodes and a pacemaker-like device to deliver pulses of electricity to specific areas of the brain. Although the mechanism of action remains to be fully elucidated, it is suggested that DBS acts via focal modulation of specific functional circuits within the brain (1, 2). The fact that the same DBS parameters and targets can benefit different neurological disorders suggests that DBS does not act against the pathophysiology of any specific disorder, but rather modulates existing and active pathologic brain circuits and it is well-tolerated (3).

The success of DBS for the treatment of Parkinson's disease (PD), in conjunction with the benefits of being adjustable, reversible, and exhibiting a good safety profile, has prompted investigation into the potential utility of neuromodulation via DBS for other diseases (4). Tens of thousands of patients suffering from different forms of neurological disorders have been treated with DBS worldwide (5), including tremor, dystonia, obsessive–compulsive disorder, depression, Tourette's syndrome, headache, chronic pain, eating disorders, and epilepsy (6). Anti-epileptic drugs (AEDs) can control seizures in most patients with epilepsy. However, at least 30% of adults with epilepsy do not achieve seizure control with AEDs (7) and surgery to remove or disconnect the epileptogenic zone is not always an appropriate option (8). These patients may be candidates for neurostimulation.

The number of potential neural targets in drug-resistant epilepsy (DRE) has increased over the years. The majority of available literature suggests targeting the anterior thalamic nucleus (ATN). In the following sections, we review the clinical outcomes for the most commonly chosen targets for the treatment of epilepsy. We included the ATN, centromedian thalamic nucleus (CMTN), hippocampus, basal ganglia (caudate nucleus, subthalamic nucleus), posterior hypothalamus and cerebellum.

## Materials and Methods

We performed a literature search of the Medline®, Embase®, Index Medicus®, Scopus, and Cochrane databases from January 1980 to October 2018 that incorporated Medical Subject Headings and text words for literature related to “deep brain stimulation” and “drug-resistant epilepsy.” We also searched bibliographies of pertinent reviews: original articles reference lists, book chapters and relevant conference proceedings to find additional documents. We included original retrospective and prospective studies assessing the impact of DBS on seizure frequency in patients with DRE regardless of language or country of origin. Children were classified as subjects younger than 18 years. Systematic reviews, meta-analyses, randomized controlled trials, and observational studies were included. We also included studies on experimental, animal or molecular models in search of an integrative review (basic and clinical science). The following outcomes were assessed: seizure reduction; seizure freedom; and time of follow-up. Discrepancies were solved by consensus. Categorical data were expressed as percentages and quantitative data as mean, standard deviation, and range. All statistical analyses were performed with SPSS statistical software package (SPSS for Mac, v.21, SPSS, Inc., Chicago, IL).

## Results and Discussion

Of the 429 abstracts identified by the search, 145 were reviewed as full-text articles. Seventy-two articles fulfilled eligibility criteria and described outcomes in 826 patients. The majority of patients were diagnosed with generalized or secondary generalized seizures (75%), while 7.2% included exclusively patients with focal seizures. Age ranged between 5 and 66 years, with a median of 30 years. All patients included in the studies had DRE.

### Historical Perspective

The beginnings of DBS date back to the late 19th century. Several authors identified the functional anatomy of the brain using animal models that went against the established beliefs and dogmas of the time (9). Horsley and Clarke (10) were pioneers in the development of stereotactic frameworks for experimental use in animals. Subsequently Spiegel et al. (11) developed the use of X-ray pneumoencephalography in 1947 allowing to visualize the living brain more accurately. As well, this enabled the creation of stereotactic atlases to guide surgeries. In 1950s we saw the introduction of neuro-ablative techniques for the treatment of Parkinsonian tremor, with the study of Albe Fessard et al. (12). They were the first to report the use of high frequency electrical stimulation (~100–200 Hz) targeting the intermediate ventral thalamic nucleus with clinical improvement in tremor severity (12). The emergence of levodopa as a highly effective pharmacological treatment for PD in the 1960s limited the development of DBS, although several authors such as Hosobuchi et al. (13) and other research groups continued their studies in other pathologies, such as chronic pain and disorders with impaired level of consciousness with encouraging results.

As further evidence accumulated through the 1980s in regards to adverse effects of levodopa, such as dyskinesias, and patients who were resistant to treatment, a better understanding of the basal ganglia (14) emerged. Furthermore, the subthalamic nucleus (STN) was identified initially as a central locus of PD (15) and therefore represented an important potential surgical target (16). This was eventually introduced into clinical practice by Pollak et al. (17). Shortly thereafter, they identified the globus pallidus interna (GPi) as another target in 1994 (18).

From these advances, the use of DBS expanded to other pathologies such as epilepsy. The first studies investigating the anti-epileptic effects of DBS in epilepsy were published in the 1970s and 1980s (19–21). Since then, numerous studies have been published evaluating the effectiveness and safety of DBS in epilepsy; however, most studies have limitations as generally report small number of patients with variable results. To date, only one large randomized control clinical trial has been published (22), generating modest evidence of benefit.

### Pathophysiology

The basic rationale for DBS as an effective anti-epileptic therapy is similar to what has been postulated for movement disorders: potential cellular inhibition or excitation (neuro-modulation) within the target structure (23). The stimulation will then either help to disrupt seizure propagation or raises the overall seizure threshold. If appropriately coordinated, low frequency stimulation (LFS) has been shown to restore normal neuronal electrical activity, while high frequency stimulation (HFS) is typically more effective at disrupting the propagation of synchronous neuronal activity (24). In hippocampal rat slice models, HFS enhances the inhibitory tone of the network and prevents the synchronization and propagation of epileptiform burst discharges (25).

**Figure 1:**
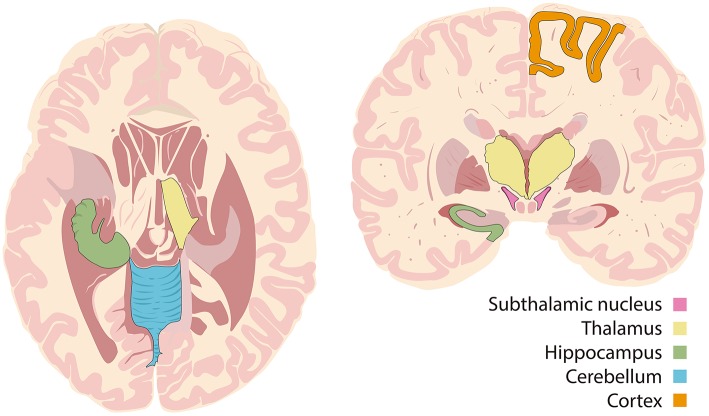
Targets of deep brain stimulation.

There are three different theories behind the selection of targets: the stimulus can be: (1) directly applied to the suspected seizure onset zone; (2) applied in deep subcortical structures to interrupt epileptic networks or; (3) applied over deeply located fiber bundles, which are connected to different structures within the brain (1, 2). The direct stimulation of the epileptogenic zone alters the tissue excitability and neuronal synchronization and can have an inhibitory effect, without causing functional deficits (1). Direct targets include the hippocampus, amygdala, hypothalamus or specific cortical zones. The indirect stimulation of deep structures may suppress neuronal circuits that favor seizure emergence. These targets include the cerebellum, basal ganglia and thalamus (2). Stimulation of fiber bundles in structures such as fornix or corpus callosum alter the threshold of seizure induction by modulation of neuronal circuits which are located in different but connected brain structures (26) (see Figure 1).

#### Networks

Papez circuit links hippocampal output via the fornix and mammillary nuclei to the ATN. Projections from the ATN then travel through the cingulum bundle to the parahippocampal cortex and complete the circuit by returning to the hippocampus. Animal studies have supported the role of this circuit as being important in seizure occurrence (27, 28). Alterations in Papez' circuit have been observed in multiple forms of epilepsy. The cerebellum, STN, and CMNT all have projections to the circuit of Papez and have therefore been considered as potentially therapeutic DBS targets for seizure reduction (29).

Another important network is the cortico-thalamo-cortical loop, which has been associated with absence epilepsy (30) and motor seizures (31) in animal models. Lesional studies in non-human primates with focal epilepsy showed almost complete suppression of seizures with thalamotomy (32). More recent studies using optogenetic techniques have shown that both thalamic and cortical neurons can trigger seizures, contrary to previous hypotheses (33–35). It has been proposed that the thalamocortical pathway acts as a “choke point” in the disruption of seizure propagation (30).

#### Basic Mechanisms

The mechanism of action underlying DBS remains poorly understood. Its action on neuronal circuits is likely multifactorial and complex. The initial DBS study suggested that its effect was inhibitory (36). This inhibition could be explained by either the blockade of depolarization and inactivation of voltage-gated currents, or, alternatively, by activation of GABAergic afferents in the stimulated nucleus (37). It is not completely clear if the therapeutic effects of DBS occur due to the stimulation of neurons, glial cells, passage fibers or afferent inputs to target neurons (38). Some studies have identified that activation thresholds were lowest in myelinated axons, and sequentially increased in unmyelinated axons, dendrites, and cell bodies (39, 40). In addition to orthodromic activation of efferent axons, multiple studies conducted in animal models and humans with PD have shown that DBS excites afferent axons in an antidromic manner (41, 42).

Other studies have shown that DBS stimulates neurons and astrocytes, producing a release of glutamate, D-serine and ATP (43). The activation of astrocytes leads to neuronal modulation through brain flow and neurovascular factors (44, 45). In addition, a “microlesion effect” has been proposed. It is demonstrated by clinical improvement in patients prior to turning the device ON, which favors the astrocytes activation hypothesis (46). Electrotaxis, the mechanism by which progenitor cells migrate through the electric current produced by DBS (47), seems to be based on inducing synthesis of growth factors and gene expression, which enhance neuroplasticity and neurogenesis (48, 49).

Neurostimulation is classified according to the method of stimulation (50). An open-loop neurostimulation system applies chronic, intermittent or continuous stimulation to inhibit epileptiform activity without reference to the patient's clinical symptoms or ongoing electroencephalogram (EEG) activity. Using an implanted seizure detection device, closed-loop stimulation provides more efficient treatment by adjusting the stimulation settings in response to EEG changes, before application of electrical stimuli (2). A burst of stimulation is applied, with the intention of terminating the detected bioelectrical change (51). Recent evidence supports the concept that closed-loop stimulation (feedback-controlled) can be more effective than open-loop therapy (52). The responsive neurostimulator system (RNS, NeuroPace, Inc., Mountain-view, CA, USA) is a prime example of a closed-loop system and has been approved by the FDA for use in epilepsy. This system applies electrical stimulation to a previously defined seizure-onset zone, triggered by detection of electrophysiological signatures of a seizure in real-time (53). RNS has demonstrated efficacy in the reduction of epileptic seizures in long-term studies, increases in quality of life scores and acceptable safety (51, 54).

## Technique

The DBS procedure consists of three steps: (1) preoperative planning, (2) surgical implantation, and (3) postoperative assessment. Prior to surgery, stereotactic coordinates for the target region are obtained by merging magnetic resonance imaging (MRI) of the patient's brain with a brain atlas (55). DBS for DRE is carried out with the patient under local or general anesthesia. The surgical procedure typically takes up to seven hours to complete and involves a multidisciplinary team of surgeons, epileptologists, and technical device staff. First, the exact placement and trajectory path for the electrode lead is determined. Next, burr holes are carefully drilled at the planned entry points for the electrodes. Region-specific neuronal activity, as a functional landmark, is used to verify the target structure during surgical procedure. One or more permanent microelectrodes are inserted into the brain using imaging guidance. Intraoperative fluoroscopy and postoperative MRI or computed tomography (CT) scans are acquired to confirm electrode placement. Finally, lead extenders are tunneled subcutaneously down the neck to below the clavicle, in which the pulse generator is implanted.

Old frame-based systems utilize a fixed frame that surrounds the patient's head entirely. New frameless systems are essentially skull-mounted aiming systems. The patient's head is registered to a scan containing the planned trajectory using intraoperative imaging, and a neuro-navigation system is then used to align the surgical trajectory with the plan. Compared to their frame-based counterparts, the new frameless systems provide benefits, such as increased patient comfort and shorter operating times. A recent American meta-analysis found a clinically insignificant loss of accuracy with frameless methods, they can therefore represent a reasonable alternative to frame-based methods (56).

### DBS Parameters

Stimulation parameters in epilepsy have been chosen empirically in the last 40 years, based on investigator experience in other pathologies, (primarily movement disorders), and center preferences. In some centers, epilepsy specialists even use electrodes that have been developed specifically for the treatment of PD (57). It is difficult to draw conclusions from existing studies suggesting parameters and predictors of efficacy, as they have been done with a small number of patients.

The evidence is scarce and often contradictory. The SANTE study found no favorable parameters for frequency, voltage, current, or pulse width after a long term follow up (22). There is no clear difference between cycling and continuous stimulation (58), or unilateral and bilateral stimulation (57). To date, usual stimulation parameters are: frequency ≥100 Hz and voltage at 1–10 V for stimulation of the ANT; frequency ≥130 Hz and voltage at 1–5 V for hippocampal and STN stimulation; HFS at voltage 1–10 V for stimulation of the CMNT; and low (10 Hz) or high (200 Hz) stimulation for the cerebellum (59). However, as there are currently no clinical randomized control trials comparing the different stimulation paradigms, the optimal parameters for epilepsy remain unknown.

Most DBS systems use a continuous, high frequency (100–250 Hz) pulse train (55). After surgery, several postoperative outpatient sessions are conducted over the course of 3–6 months, by a clinician who optimizes stimulation parameters based on patient feedback and seizure control (60). Often the clinician is a neurologist or an epilepsy nurse, who determines the optimal parameters including amplitude, frequency and pulse width.

## Targets of Stimulation

### Thalamus

#### Anterior Thalamic Nucleus (ATN)

The ATN is the most widely used target for DBS in treatment of DRE (22). It has been preferred because of its size, its distance from vascular structures (24), and its extensive connections. Several studies have indicated that the anterior thalamic region is crucial to the maintenance and propagation of seizures (61). This is explained by its connections to the limbic system through the fornix and mammillothalamic tract with widespread and extended projections to the cingulate, entorhinal cortex, hippocampus, orbitofrontal cortex, and caudate, all of which have been implicated in the pathogenesis of focal epilepsy (62).

Small open-label, uncontrolled, pilot studies have shown clinical benefits. A 49% reduction of seizure frequency was reported in four patients after 44 months of follow-up (58). Similarly, a mean reduction of 75% was observed in four patients with mesial temporal lobe epilepsy (TLE) after treatment with DBS-ATN (63). Lee and colleagues investigated 15 patients with DRE, who underwent placement of bilateral DBS electrodes in the ATN. They showed a significant decrease (70%) in seizure frequency and it was concluded that the short-term outcome of ATN-DBS directly reflects the long-term outcome (64). Additionally, Kerrigan et al. (65) reported four out of five patients with significant reductions in the frequency and severity of seizures after a 36 month follow-up, without any complications. Hodaie et al. (61) reported a mean reduction of 54% in seizure frequency during a 15 month follow-up, also without adverse effects. Finally, Andrade et al. (66) described five of six patients (83%) with at least 50% improvement in seizure frequency over a mean follow-up period of 5 years. Sleep disruption and neuropsychiatric symptoms have been reported as a voltage-dependent adverse effect of DBS in ATN in patients with epilepsy (67).

These studies led to a randomized clinical trial in 2010 called *Stimulation of the Anterior Nucleus of the Thalamus for Epilepsy* (SANTE) (22). This study was a multicenter, double blind, clinical randomized control trial investigating the use of bilateral DBS of the ATN for the treatment of localization-related epilepsy. One hundred and ten patients with focal or secondarily generalized seizures, intractable to drug treatment, were divided into two groups. Half of the participants received stimulation, and half of the patients received no stimulation over a 3-month blinded phase. Subsequently, all patients received un-blinded stimulation. This trial reported significant improvement in seizure frequency, especially in focal seizures with altered awareness and severe seizures after 25 months of follow-up. Of the 110 patients initially enrolled in the study, 81 (74%) completed the follow-up period. Among these patients, the median decrease in seizure frequency was 56%, ranging widely from a 26% increase in frequency to complete seizure freedom (six patients). Median seizure frequency reduction continued to improve over the 3 years of the trial, with a 41%, 56%, and 68% median seizure frequency reduction at 1 year, 2 years, and 3 years of DBS, respectively, with 29% greater reduction in seizure attacks in the stimulated group compared to the control group, observed during the last month of treatment (22).

No surgery-related symptomatic hemorrhages or deaths were reported, although two participants had transient, stimulation-induced seizures. Other adverse events included paraesthesias at the implant site in 18%, local pain in 11%, and infection in 9% of cases. Depression and memory impairment were more frequent in the stimulated group compared with the controls. Patients with temporal lobe seizures showed a greater benefit during the blinded phase compared with those with extratemporal or multifocal seizure onsets (62). Some studies have suggested that bitemporal mesial epilepsy may be the most responsive to ATN stimulation (22). Direct targeting in the ATN using high-resolution MRI is likely superior to indirect targeting due to extensive individual variation in the location of the ATN and may therefore improve the efficacy of DBS (68). Furthermore, performance of ATN-DBS parameters with simultaneous EEG recording during the ATN-DBS has been suggested to improve the therapeutic efficacy by monitoring of EEG desynchronization (69). As demonstrated in previous studies, DBS had a better effect over time.

### Centromedian Nucleus of Thalamus (CMNT)

Dense cluster of axons project from CMNT, a midline thalamic structure, to the dorsolateral part of putamen. The CMNT also projects to the cerebral cortex, principally to the motor and premotor cortices (70). Anatomical patterns of CMNT connections support its role in the pathophysiology of generalized seizures. Animal studies have demonstrated the CMNTs role in the initiation of seizures (71, 72) as well as in improvement of level of postictal consciousness after stimulation of the CMNT (73).

Stimulation of the CMNT in humans for treatment of DRE was first performed by Velasco and colleagues (74). CMNT stimulation appears to be more suitable for the control of absence and generalized seizures, especially in patients with primary or secondary Lennox Gastaut syndrome (LGS) with up to 80% of patients showing a good response. It does not appear to be effective for the treatment of focal seizures with altered awareness (74). Targeting the parvocellular division of the CMNT bilaterally, Velasco et al. (75) observed a reduction in seizure frequency in 13 patients with LGS. However, this was an open-label uncontrolled case series. In the only controlled pilot study of CMNT stimulation, preformed in seven patients, Fisher and colleagues found a 50% reduction in seizure frequency in three patients, treated with 24 h/day continuous stimulator trains (76).

Eleven patients with generalized or frontal lobe DRE epilepsy were recruited at King's College Hospital (London, United Kingdom) and at the University Hospital La Princesa (Madrid, Spain) (77). They underwent bilateral DBS targeting the CMNT. Among the eleven patients, seven (64%) demonstrated improvement. Among the five patients with frontal lobe epilepsy, only one patient (20%) had significant improvement (more than 50% of reduction in seizure frequency) during the blind period; and two (40%) during the long-term extension phase. However, all six patients (100%) with generalized epilepsy had significant improvement in seizure frequency during the blind period; and in the long-term extension phase, five of the six (83%) patients showed more than 50% improvement in the frequency of seizures. Among patients with generalized DRE epilepsy, DBS implantation and stimulation of the CMNT appeared to be effective and safe. One patient (9%) had the device removed 6 months after implantation due to infection and one patient (9%) reported a transient agraphia in the first 4 days following implantation. Improvement in seizure attacks was observed during months 3 to 50 with the DBS device turned OFF (77). Additional large and well-controlled studies for CMNT stimulation are needed to identify the efficacy, mechanism and the target population (Table 1).

**Table 1:** Clinical studies of Bi-ATN and CMT-DBS for the treatment of Epilepsy.

**Author/year**	**Mean age (years)**	**Design**	**n**		**Seizure type (s)**	**Follow up (months)**	**Average seizure reduction (range)**
**BI-ATN**							
Upton et al. (21)	24	Open label	6		CPS	>36	4/6 had “significant clinical control”
Sussman et al. (78) (Abstract)	NR	Open label	5		CPS, SGTC	12–24	60% showed “improvement”
Hodaie et al. (61)Andrade et al. (66)	30	Single blind	5 (+1)		GTC, DA, CPS, AA, SGTC, PM	4–7 years	55% (24–89%)
Kerrigan et al. (65)	36	Open label	5		SPS, CPS, SGTC	20.4 (6–36)	48% (−57–98%) of “serious seizures”
Lee et al. (79) (& Bi-STN)	22	Open label	3		TS, DA, HM, AM, SGTC	6 (2–10)	75.4% (50–90.6%);3/3 RR
Lim et al. (58)	27	Open label	4		G, P, STGC	24	49% (35–76%);1/4 RR
Osorio et al. (63)	31	Single blind	4		(Bi- MTLE)SGTC, CPS, SPS, DA	36	75.6% (53–92%);4/4 RR
Andrade et al. (80)	29, 45	Open label	2		(DRA)SGTC, MYO, CS, GTC	120	98% of SGTC in one; 66% total in other
Fisher et al. (22) (SANTE)	36	Clinical trial (Double blind, randomized, parallel group)	54	55^*^	CPS, SGTC	24	26% above controls after 2 months; 56% median reduction after 2 years
Lee et al. (64)	31	Open label	15		CPS, GTC, SPS	39 (24–67)	70.5% (0–100%);13/15 RR
Oh et al. (81)	33	Open label	9		CPS, SGTC	34.6 (22–60)	57.9% (35.6–90.4%);7/9 RR
Van Gompel et al. (82) (& Bi-HC)	26, 32	Open label	2		SPS, CPS, SGTC	3	80% in one; 53% in other;2/2 RR
Piacentino et al. (83)	38	Open label	6		(LGS), CPS, SGTC	>36	3/5 RR^**^
Voges et al. (67)	37	Case-Cohort study, Open label	9		CPS, SGTC	28	7/9 RR
Lehtimaki et al. (84)	35	Open label	15		NR	25.2 (9–52)	10/15 RR
Salanova et al. (85) (SANTE)	NR	Clinical trial	83		SANTE study	61	69%
Krishna et al. (86)	32	Open label	16		SPS, CPS, SGTC, GTC, MYO, DA	52	65% (−500–99%) at 3 y;11/16 RR
Franco et al. (87)	51, 48	Open label	2		(SBH)CPS, SGTC	18, 12	61% in one, 75% in other;2/2 RR
Valentin et al. (88)	15	Open label	1		SPS	12	>60%
Nora et al. (89)	30	Open label	1		GTC	40	87%
Piacentino et al. (90)	48	Open label	1		CPS, GTC	60	100%^***^
Jarvenpaa et al. (91)	38	Open label	16		NR	24	12/16 RR
**CMNT**							
Velasco et al. (92)	18	Open label	5		GTC, CPS, MYO, DA	6–37	80–100% GTC;60–80% CPS
Fisher et al. (76)	28	Clinical trial (Double blind, cross over)	6		GTC	9	30%
Velasco et al. (74)	19	Clinical trial (Open label)	13		(LGS)AA, GTC, CPS, SGTC	41.2 (12–4)	81.6% (53.1–100%) LG;57.3% (13–98.6%) SGTC
Chkhenkeli et al. (93) (& HCN, CDN)	21–40 range	Single blind	5 of 54		SPS, GTC, CPS, SGTC, TS, PM	≤18	4/5 “worthwhile improvement”;1/5 no improvement^¥^
Velasco et al. (75)	13	Open label	13		(LGS) AA, GTC	46 (23–132)	80% (30–100%)
Andrade et al. (66)	NR	Open label	2		GTC, SPS, CPS, SGTC	≤7 years	0/2 RR
Cukiert et al. (94)	29	Open label	4		DA, AA, MYO, TS, TC, TA	18 (12–24)	80% (65–98%)
Valentin et al. (95)	27	Open label	1		RSE	6	100%
Valentin et al. (77)	37	Single blind	11		G or FLE	24 (12–66)	82% (40–100%) G;49% (0–92%) FLE;6/6 RR in G;1/5 RR in FLE
Son et al. (96)	29	Open label	14		(LGS) SPS, CPS, GTC, G, DA, MYO, AA	18.2	68% (25–100%);11/14 RR
Valentin et al. (88)	10, 8	Open label	2		GTC, TS, TA, DA, MYO	48, 18	>60% in one; no significant reduction in other

***Seizure types:** G, Generalized; P, Partial; CS, Clonic; SPS, Simple partial seizure; CPS, Complex partial seizure; DA, Drop attack: atonic; DIA, Dialeptic; GTC, Generalized tonic-clonic; AA, Atypical absence; SGTC, Secondarily generalized; PM, Partial motor; TS, Tonic; TC, Tonic-clonic; TA, Typical Absence; HM, Hypermotor; AM, Automotor; MYO, Myoclonic; RSE, Refractory status epilepticus; DRA, Dravet Syndrome; SBH, Subcortical band heterotopia; LGS, Lennox-Gastaut Syndrome*.

***Targets:** ATN, Anterior thalamic nucleus; CMNT, Centromedian nucleus of thalamus; HCN, Head of caudate nucleus; CDN, Cerebellar dentate nucleus*.

***Seizure onset:** Bi, Bilateral; Uni, Unilateral; TLE, Temporal lobe epilepsy; MTLE, medial temporal lobe epilepsy; FLE, Frontal lobe epilepsy*.

***Outcomes:** RR: Responders rate (Seizure reduction ≥50%); Serious Seizures: Potentially injurious seizures (SGTC or CPS associated with falls)*.

***Other:** NR, Not report*.

* *SANTE trial control patients (22)*.

** *One died, not related to DBS (83)*.

*** *The patient had long-term significant reduction in seizure frequency even with an absent electric stimulation (90)*.

¥ *Engel Classification (93)*.

### Hippocampus

TLE is the most frequent focal epilepsy syndrome in humans and is frequently associated with hippocampal sclerosis and DRE. Temporal lobe resection is the optimal therapy for patients with refractory mesial TLE (97). Unfortunately, this is contraindicated in a considerable number of patients, including those whose seizures originate in both temporal lobes, those at risk for a postoperative decline in memory, cognitive function and language, and those who have had a previous temporal lobectomy but continue to have seizures originating from the contralateral temporal lobe (98).

Twenty years ago, hippocampal LFS [2-Hz pulses −500 μA base to peak, 1 ms duration biphasic square-wave pulses] was used to trigger seizures in experimental models (99). Recently, studies done in rat models of TLE have demonstrated that LFS applied at a frequency of 1 Hz, significantly reduced the excitability of the neuronal tissue, resulting in decreased seizure frequency (100). Remarkably, hippocampal HFS can protect hippocampal neurons against kainate neurotoxicity in macaques, likely via the inhibition of apoptosis (101).

Continuous electrical stimulation, especially with high frequencies (130 Hz), has been shown to completely inhibit picrotoxin- and high-K+-induced epileptiform activity in animal *in vivo* models of epilepsy (102, 103). Several clinical investigations support the anti-seizure effect of electrical stimulation of the hippocampus. Direct stimulation over the suspicious epileptogenic zone within the hippocampus is applied in this procedure. Two studies describe a decrease in interictal epileptiform discharges (93, 104). Téllez-Zenteno and his group reported a 15% seizure frequency reduction in four patients after unilateral DBS in the hippocampus (98). Boon et al. (105) studied the effect of DBS in medial temporal lobe in 10 patients with DRE. After mean follow-up of 31 months, one patient was seizure free, one demonstrated a more than 90% reduction in seizure frequency; five of 10 patients had ~50% seizure-frequency reduction; two had a reduction of 30–49%; and one remaining patient was a non-responder, with no change in seizure frequency. No serios clinical adverse effects (except an asymptomatic intracranial hemorrhage in one patient) or alterations in clinical neurological testing have been reported. In another study, McLachlan et al. (106) studied the effect of continuous bilateral electrical stimulation of the hippocampus in two patients with seizures originating from bilateral mesial temporal lobes. Seizure frequency decreased by 33% in both patients during stimulation and remained 25% lower for the 3 months after stimulation was turned off. No consistent changes were seen in objective or subjective measures of memory. No other adverse effects were reported. More surprisingly, Vonck et al. (107) described three patients with neuromodulation of the amygdala-hippocampal junction who exhibited a 50–90% decrease in seizures. The larger prospective, controlled, double-blind study evaluating the effects of Hippocampus-DBS in 16 TLE patients has shown that HFS (130 Hz) is effective in reducing seizure frequency in patients with refractory TLE. Fifty-percent of this cohort became seizure-free (108).

To evaluate anatomical and functional changes in amygdala-hippocampal function after DBS in TLE patients, several studies have been conducted. Velasco et al. (109) found that by extending the period of follow-up from 18 months to seven years, patients could be divided into two groups: patients with normal and abnormal MRI studies. Ninety five percent of patients with non-lesional epilepsy exhibited more than 95% of improvement, while only 50–70% of patients with hippocampal sclerosis identified on MRI showed improvement with DBS. None of these patients exhibited neuropsychological deterioration. Micro-lesions have been reported in a few epileptic patients, following the diagnostic implantation of depth electrodes in the temporal lobe (110, 111). Altogether, DBS appears to be a safe and valuable option for patients who suffer from drug-resistant TLE in whom resective surgery is contraindicated. Hippocampal DBS has also been found to be a relatively safe procedure. No irreversible cognitive or psychiatric deficits were encountered (112). However, even with the potential benefits, hippocampal stimulations cannot be considered as a first line therapy, in lieu of a resective procedure (Table 2).

**Table 2:** Clinical studies of HC-DBS for the treatment of Epilepsy.

**Author/year**	**Mean age (years)**	**Type of study**	**n**	**Seizure type (s)**	**Follow up (months)**	**Average seizure reduction (range)**
**HC**						
Velasco et al. (113, 114)	24	Open label	10	(TLE) CPS, SGTC	2 weeks	100% after 6 days
Vonck, 2002 (115)	33	Open label	3	(MTLE) CPS, GTC	5 (3–6)	77% (50–94%)
Vonck et al. (116)	NR	Open label	7	(TLE)NR	14 (5.5–21)	43% (0–100%)
Tellez-Zenteno et al. (98)	32	Clinical trial (Double blind, cross over)	4	(MTLE)CPS, SGTC	6 blind	26% (ON) vs. −49% (OFF)
Boon et al. (105)	NR	Open label	10	(MTLE)CPS, SPS, SGTC	31 (15–52)	50% (< 30–100%)
Velasco et al. (109, 117)	29	Clinical trial	9	(MTLE)CPS, SGTC	18 (1 blind)	83% (50–100%);9/9 RR
McLachlan et al. (106)	45, 54	Clinical trial(Double blind, cross over)	2	NR	3	33% (ON) vs. 4% (OFF)
Boex et al. (110)Bondallaz et al. (118)	34	Open label	8	(MTLE) CPS, SGTC	44	67% (0–100%);6/8 RR
Tyrand, 2012 (119)	32	Open label	12	(TLE)NR	0	58.1%^*^
Morrell et al. (53) (RNS trial)Heck et al. (54) (RNS trial)	34.9 (18–66)	Clinical trial	95 of 191	SPS, CPS, SGTC	3 blind 48	38% (ON) vs. 17% (OFF)53%,55% RR
Vonck et al. (107)	NR	Open label	11	(MTLE)CPS, SPS, SGTC	96 (67–120)	70% (0–100%);8/11 RR;
Cukiert et al. (57)	37	Single blind	9	(TLE) CPS, SPS, SGTC	30.1	61% (−50–100%); 7/9 RR
Jin et al. (120)	NR	Open label	3	CPS, SGTC	35	93% (91–95%)
Lim et al. (121)	35	Open label	5	CPS, SGTC	38	45% (22–72%); 3/5 RR
Cukiert et al. (108)	38.4	Clinical trial (Double blind, randomized)	16	SPS, CPS	8 (6 blind)	3/14 RR in CPS, 7/16 RR in SPS

***Seizure types:** P, Partial; CS, Clonic; SPS, Simple partial seizure; CPS, Complex partial seizure; DA, Drop attack: atonic; DIA, Dialeptic; GTC, Generalized tonic-clonic; AA, Atypical absence; SGTC, Secondarily generalized; PM, Partial motor; TS, Tonic; HM, Hypermotor; MYO, Myoclonic; PME, Progressive myoclonic epilepsy*.

***Targets:** ATN, Anterior thalamic nucleus; VIM, Ventral intermediate nucleus of thalamus; STN, Subthalamic nucleus; HC, Hippocampus*.

***Seizure onset:** Bi, Bilateral; Uni, Unilateral; TLE, Temporal lobe epilepsy; MTLE, medial temporal lobe epilepsy; FLE, Frontal lobe epilepsy*.

***Outcomes:** RR: Responders rate (Seizure reduction ≥50%)*.

***Other:** NR, Not report*.

* *Outcome: Reduction of interictal activity with biphasic stimulation in Hippocampal sclerosis (119)*.

### Basal Ganglia

#### Caudate Nucleus (CN)

The CN may represent a deep target for the treatment of epilepsy. Low frequency electrical stimulation has been found to be efficacious when targeting the caudate (112). Interestingly, high frequency (30–100 Hz) stimulation of the head of the caudate nucleus (HCN) caused enhancement of epileptiform spikes from the ipsilateral hippocampus and amygdala while, on the contrary, LFS of the caudate reliably produced inhibitory effects bilaterally. The caudate loop is a functional entity comprised of the HCN, thalamus and neocortex. Activation of HCN is associated with hyperpolarization of cortical neurons, suggesting that suppression of seizure activity may be a result of stimulation-induced inhibition of the cortex (122).

Trials studying the effect of caudate stimulation on patients with epilepsy have not been systematically conducted and have yielded only marginal results. Sramka and Chkhenkeli (123) published a study of 74 patients showing reduced interictal epileptiform activity with both caudate and dentate nucleus stimulation. Chkhenkeli et al. (93) stimulated the ventral HCN at low frequency (4–8 Hz) in 38 patients, producing seizure freedom in 21 patients, while improving 35 patients overall (92%). This group was the first to describe benefit with striatal stimulation in cases of drug-resistant TLE. Their study was based on a pathophysiological hypothesis related to the balance of output between pro-convulsant and anti-convulsant structures, however, their work suggests that LFS of the CN is anti-epileptic (124). These results highlight the ability of the basal ganglia to modulate cortical epileptogenicity. Controlled clinical studies are necessary to determine efficacy and safety of this anatomical location. Currently, the CN is not the most frequently targeted structure in the treatment of DRE.

#### Subthalamic Nucleus (STN)

STN DBS has been explored as an option to treat motor seizures through the disruption of pathological cortical synchronization. Inhibition of the STN may potentially release the inhibitory effect of the substantia nigra pars reticulata on the dorsal midbrain anticonvulsant zone, thus raising the seizure threshold. This mechanistic rationale has arisen from observations in animal models (125, 126). The compact and distinct anatomical structure of the STN makes it a superior surgical target for electrode stimulation, which has been previously demonstrated in DBS-STN for treatment of PD.

Benabid et al. (127) reported a series of three patients with DRE who were implanted with STN-DBS. All patients were reported to exhibit important reductions in seizure frequency with stimulation; the first two patients exhibited reductions of 83 and 50%. Afterwards, the group reported a case describing a 5-year-old girl with DRE caused by focal centroparietal dysplasia, followed for 30 months. She had a 81% improvement in the number of seizures. This was most substantially reflected in reduction in cluster seizures (89%) and diurnal seizures (88%) (128).

Small studies have been conducted in severely impaired patients with severe DRE. Chabardes et al. (129) demonstrated a mean of 60% seizure frequency reduction in 80% of patients (4/5). They included patients aged from 5 to 38 years (17.6 ± 12.7). The stimulation was well-tolerated and appeared to demonstrate efficacy, however there were some complications related to the procedure in 40% (2/5) of patients; namely, a device infection in one patient, and a post-implantation subdural hematoma in a second patient, requiring re-operation.

Forty percent (2/5) of patients who underwent STN-DBS at the Cleveland Clinic Foundation reported a seizure rate reduction of 60% at 10 months of follow-up and 80% at 16 months (130). STN/substantia nigra DBS resulted in 30–100% reduction in seizure frequency of five patients with drug-resistant myoclonic epilepsy (131). STN-DBS may be a favorable target in certain epilepsy syndromes, but controlled, blinded trials are required to demonstrate efficacy and safety.

### Cerebellum

The cerebellar nuclei have been some of the oldest targets in DBS, with initial uncontrolled trials in the 1970s (20) demonstrating potential efficacy in the treatment of epilepsy. Nuclear activation of inhibitory Purkinje cells, likely results in the suppression of excitatory cerebellar output to the thalamus and therefore, decreased excitatory thalamo-cortical projections, resulting in overall decreased cortical excitability (132). Disrupting thalamo-cortical activity has proven to be a useful approach to stop generalized spike-and-wave discharges in mice. It has been demonstrated that cerebellar nuclei are modulators of pathological oscillations during absence seizures (133).

Seventy six percent of epileptic patients (87/115) treated with cerebellar nuclei modulation demonstrated benefit with a reduction in seizure frequency. Overall, 27% reported seizure freedom and 49% reported reduction in seizure frequency and severity. Those patients with generalized tonic-clonic seizures benefited the most (134).

Velasco et al. (135) conducted a double blind, randomized control pilot study with five DRE patients with motor seizures. They implanted stimulating electrodes on the supero-medial cerebellar cortex and evaluated the efficacy and safety. After 6 months of stimulation, all patients reported a seizure rate reduction, on average 41% (14–75%) compared with the control group. At the end of 24 months, the three (60%) patients who completed follow up reported a further seizure reduction of 24% (11–38%) (135). In regards to the safety profile of the intervention, 60% of patients required another procedure owing to electrode migration, and 20% (one patient) suffered a severe local infection that finally resulted in long-term antibiotic therapy and removal of the entire stimulation system (136). After these preliminary studies, cerebellar stimulations have not been used in recent studies.

### Hypothalamus

Due to the presence of epileptiform activity during depth electrode recordings of the mammillary bodies, the posterior hypothalamus has been suggested as a DBS target (137). The mammillo-thalamic tract stimulation has been used to treat gelastic seizures secondary to hypothalamic hamartomas, and has shown an improvement in seizure frequency and severity (138). A report of two patients by Franzini et al. (139) showed a reduction up to 80% in seizure frequency from baseline after 9 months of follow-up. This target may be unfavorable due to consequences of potential hemorrhage in this region during electrode implantation, as well as possible alterations in sleep-wake cycle (140). A recent study targeting the posteromedial hypothalamus reported nine patients with DRE, associated with intractable aggressive behavior, achieved a significant decrease in the frequency of epileptic seizures after up to 5 years of follow-up, achieving an average seizure reduction of 89.6% (141). Current experience with hypothalamic stimulation is too limited to draw firm conclusions. Other targets such as the caudal zona incerta, nucleus accumbens and fornix have been also explored (see Table 3).

**Table 3:** Clinical studies of STN, Cerebellum (CB), HNC, CZI, pHT, NA, and Fornix -DBS for the treatment of Epilepsy.

**Author/year**	**Mean age (years)**	**Type of study**	**n**	**Seizure type (s)**	**Follow up (months)**	**Average seizure reduction (range)**
**STN**						
Benabid et al. (128)	5	Open label	1	SPS	30	80.7%
Chabardes et al. (129)	18	Open label	5	TS, CS, GTC, HM	18 (8–30)	51.4% (0–80.7%)
Shon et al. (142)	23, 22	Open label	2	(FLE) TS	18, 6	86.7% in one;88.6% in other
Handforth et al. (143)	45, 46	Open label	2	P	26–32	50% and 33%
Lee et al. (79) (& ATN)	20	Open label	3	DIA, SGTC, TS	18, 30, (1 loss)	49.1% (20–71.4%)
Vesper et al. (144)	39	Open label	1	(PME), GTC, MYO	12	50% MYO,100% GTC
Wille et al. (131) (& VIM)	32	Open label	5	(PME), GTC, MYO	24 (12–42)	30–100%
Capecci et al. (145)	35, 30	Open label	2	PM, GTC, DA, CPS, AA	48, 18	65% in one and 0% in other
**CB**						
Cooper et al. (20)	29	Open label	15	CPS, SGTC, GTC, MYO, TA	27	10/15 “improved”
Van Buren et al. (19)	27	Double blind, cross over	5	CPS, SGTC, GTC, MYO	15–21 range	No significant reduction
Levy et al. (146)	29	Open label	6	GTC	7–20 range	2/6 RR
Bidznski et al. (147)	NR	Open label	14	NR	10–16 days	13/14 “improved”; 1/14 no change
Wright et al. (148)	30	Clinical trial (Double blind, cross over)	12	GTC, DA, A, MYO, CPS	6 blind	No statistically significant; 11/12 patients felt it helped
Davis et al. (149)	NR	Open label	27	Spastic seizures	17 years	23/27 improved; 4/27 no improvement
Chkhenkeli et al. (93) (& HCN, CDN)	21–40 range	Single blind	11 of 54	GTC, CPS, SGTC, TS, PM	≤18	5/11 seizure free;5/11 “worthwhile improvement”;1/11 no improvement^*^
Velasco et al. (135)	26	Clinical trial (Double blind, cross over)	5	GTC, TS, DA, MYO, AA	24 (3 blind)	67% (ON) vs. 7% (OFF);76% (62–89%) GTC; 57% (24–90%) TS
**HCN**						
Chkhenkeli et al. (124)	NR	Open label	57	NR	NR	Unclear
Chkhenkeli et al. (93) (& CDN)	NR	Open label	38 of 54	GTC, CPS, SGTC, TS, PM	≤18	21/38 Seizure free;14/38 “worthwhile improvement”;3/38 no improvement^*^
**CZI**						
Franzini et al. (139) (& pHT)	26	Open label	2	(RS)SPS, SE	6, 48	85% in one, andremission of SE in other;2/2 RR
Anderson et al. (150)	20	Open label	3	(NSPM)GTC, MYO, TA	4.3 years (3–6)	3/3 “improved”
**pHT**						
Franzini et al. (139)	20, 36	Open label	2	DA, MYO, CPS	9, 60	75% in one and 80% in other;2/2 RR
Benedetti et al. (141)	21	Open label	5	SPS, CPS, GTC, AA, DA,	5 years	89.6% (25–100%);5/5 RR
**NA**						
Schmitt et al. (151)	42	Open label	5	SPS, CPS, GTC	6	37.5% median; no significant changes in mean frequencies;2/5 RR of DS
Kwoski et al. (152)	37	Clinical trial (double blind, cross over)	4	SPS, CPS, SGTC	15 (6 blind)	17.2% (ON) vs. −1,6 (OFF) of DS at 28 days;3/4 RR of DS
**FORNIX**						
Koubeissi et al. (26)	41	Open label	7	(MTLE), SPS, CPS	1–9 days	Seizure odd reduced by 92% for day 1–2

***Seizure types:** SPS, Simple partial seizure; CPS, Complex partial seizure; TA, Typical Absence; GTC, Generalized tonic-clonic; SGTC, Secondarily generalized; SE, Status epilepticus; DA, Drop attack: atonic; AA, Atypical absence; MYO, Myoclonic; PM, Partial motor; TS, Tonic; RS, Rassmusen syndrome; NSPM, North Sea Progressive Myoclonic Epilepsy*.

***Targets:** CB, Cerebellum; HCN, Head of caudate nucleus; CZI, Caudal zona incerta; pHT, Posterior hypothalamus; NA, Nucleus accumbens; CDN, Cerebellar dentate nucleus*.

***Seizure onset:** Bi, Bilateral; Uni, Unilateral; MTLE, medial temporal lobe epilepsy*.

***Outcomes:** RR: Responders rate (Seizure reduction ≥50%); DS: Disabling seizures (CPS+ GTC)*.

***Other:** NR, Not report*.

* *Engel Classification (93)*.

## Adverse Effects

There are well-known side effects and potential complications associated with DBS, which have been mainly elucidated by the literature regarding DBS in the treatment of movement disorders (153). The overall complication rate for DBS surgeries in patients with PD was 7%, which included mechanical complications (3%), hemorrhage or infarction (1%), lead removal (1%), hematoma (0.4%), and infection (0.4%) (154). Comparatively limited information is available regarding the specific complications of DBS in patients with epilepsy. The most common stimulation-related side effects in the SANTE studies were mainly those expected from implanted electrodes, including stimulation-related paresthesias (22.7%), implant site pain (20.9%), implant site infection (12.7%), and subclinical bleeding around electrodes (22). Other known complications include: wound infection; lead or extension fracture; erosion; lead tract fibrosis; electrode migration; external interference with other devices; equipment infection; pain; transient worsening or new seizures; and dizziness (59); skin complications [such as abrasions, ulcerations and aseptic necrosis (155)]; hardware discomfort; ineffective product (85, 156); and peri-electrode edema (157) that may produce disorientation, gait instability, headache, seizure or acute confusion (155).

### Psychiatric Side Effects

#### Depression and Suicide

A large variability in the use of diagnostic scales measuring depression is noted in the existing literature regarding DBS. It is therefore difficult to directly compare the results of the studies. Anterior thalamic DBS was initially reported to be associated with higher rates of memory deficits and depression (158); however, 5-year (85) and 7-year follow-up (156) of the SANTE study population found no significant deterioration in cognition or depression scores. Randomized multicenter and observational studies have shown improvement in depression and anxiety scores after DBS in patients with PD (159, 160). However, higher prevalence of depression after STN-DBS has also been reported (158, 161). PD patients treated with STN-DBS were also found to have a higher suicide rate, in a paper published a decade ago. Suicidal behavior was frequently associated with postoperative depression and altered impulse regulation (162, 163). A recent randomized controlled trial did not find a direct association between suicide and DBS (164). This may be indicative of improved patient selection criteria in recent years. This is further complicated by the fact that suicide after DBS has occurred not only with different anatomic targets (particularly with thalamic and GPi stimulation) but also with other diseases (dystonia and essential tremor) (165). Although the evidence is contradictory and scarce in epilepsy, all patients should be carefully screened for suicide risk as part of the presurgical workup for DBS surgery. Additionally, patients should be monitored closely for depression and suicidality post-operatively.

#### Apathy

Apathy has been frequently reported as a possible adverse effect of STN-DBS, however the existing literature is controversial. Some authors have found significant worsening of apathy scores 3–6 months after surgery (166). They hypothesized a direct influence of STN-DBS on the limbic system by diffusion of stimulus to the medial limbic compartment of STN. Recently, a metanalysis assessing apathy following bilateral DBS of the STN in PD concluded that the reduction of dopaminergic medication after surgery may be the cause of worsening apathy in this patient population (167). Other authors have failed to find significant differences in apathy prevalence or severity between surgical and non-surgical patients (168). On the contrary, some publications have stated positive psychiatric side effects of DBS (169). Results of STN stimulation in patients with PD in a Polish study confirmed the positive effects of stimulation on drive and ability to feel pleasure. The authors demonstrated improvement of mood, sleep and apathy following the first month after initiation of stimulation, which was seen independently of improvements in motor symptoms (170).

#### Cognitive Deterioration

Cognitive decline reported after DBS mainly affects frontal subcortical cognitive functions, such as verbal fluency, processing speed, attention, learning, and working memory (171). Worse cognitive outcomes after surgery remained unchanged, regardless of DBS settings or “on” and “off” motor states, suggesting the cause might be related to lead trajectory or location (172). Some psychiatric side effects have been also described, such as psychosis (for example, delusions of marital infidelity resembling Othello syndrome), and impulse control disorders, such as binge eating, hypersexuality, hypomania, and secondary increase in body weight (155).

### Other Side Effects

Other less mentioned and more infrequently encountered, but nonetheless important complications include post-surgical headache, instability and gait disorders with falls (170, 173), as well as speech disorders (dysarthria, intelligibility, pitch variation in speech, worsening hypophonia, stuttering, and speech articulation problems) (155, 174). When the device is less frequently used, the complications are higher, for instance, bilateral cerebellar stimulation has been associated with re-operation in 60% and serious complications in 20% of the patients (136).

## Epilepsy Comorbidities

People with epilepsy in the general population have a two to five-fold risk of somatic comorbid conditions compared to people without epilepsy. Thus, there is a clear need for an integrated approach in patients with epilepsy, especially in those with DRE who are good candidates for electrical stimulation. The process of localizing DBS targets is undergoing continuous evolution. The clinical effects of DBS are likely due to the activation of complex, widespread neuronal networks, directly and indirectly influenced by the stimulation of a single isolated target. The delivery of such stimulation may aid in the discovery of strategically combined targets for electrical stimulation to treat additional neurological, psychiatric, cognitive and somatic disorders. Computational modeling (experimental and clinical), engineering designs, and neuroimaging techniques play a critical role in this process (175).

### Depression

Depression is the most common comorbidity of epilepsy, affecting between 10% to 60% of patients with seizures (176). Different targets have been used in patients with refractory major depressive disorder, such as subcallosal cingulate gyrus (Brodmann area 25), ventral capsule/ventral striatum, medial forebrain bundle, and the nucleus accumbens. The overall effect sizes have shown a significant reduction in Hamilton depression rating scale scores after DBS stimulation in these four regions (177). Selected patients with refractory depression and DRE could benefit from stimulation of these targets.

### Obesity

There is some evidence that obesity may be more common in people with epilepsy than in the general population (178). The pathophysiology of obesity is complex, involving both altered patterns of eating and satiety, as well as compulsive behavior surrounding food intake. Proposed stimulation targets to treat obesity therefore include the hypothalamus and nucleus accumbens (179). To date, experience with DBS for the treatment of obesity is limited. Although surgery has been proven to be safe, no definitive conclusions can be made as to whether it is effective. There is an opportunity to study this further in patients with epilepsy and obesity, especially in those with gelastic seizures secondary to hypothalamic hamartomas. Further work is needed to address target selection, the kinetics of DBS, and the ideal stimulation parameters in this population.

### Psychogenic Non-epileptic Seizures

Psychogenic non-epileptic seizures (PNES) are one of the most common differential diagnoses of epilepsy. PNES are involuntary episodes of any combination of altered movement, sensation, or awareness that bears resemblance to epileptic seizures, but are not accompanied by epileptiform electrical discharges (180). Unfortunately, PNES are an exclusion criterion for DBS candidates in most of the epilepsy centers. There are few reports of the experience with psychogenic movements disorders and DBS. This literature suggests that DBS does not produce side effects in patients with psychogenic disorders. One patient with psychogenic parkinson-like disorder underwent HFS of the STN for approximately 5 years without side effects, prior to the device being turned off (181). Similarly, authors from Bethesda described two cases of psychogenic dystonia who underwent DBS in the GPi, after initially being thought to have organic dystonia (182). DBS did not lead to any benefits or side effects for these patients. These reports further highlight the safety of DBS in neuronal tissue.

## Limitations

Many of the available publications are non-randomized, unblinded, uncontrolled small studies. Therefore, the evidence is susceptible to major biases:

- Regression to mean: patients are typically implanted when there is high seizure burden. In all chronic diseases, phases of high activity and lower activity are observed, thus seizure burden may have returned to baseline without any therapy.
- Placebo effect: even in some controlled studies, there is no “sham-surgery” arm that would allow for estimation of the effect of an invasive procedure on the subjective seizure assessment.
- High expectations of both investigator and patients, resulting highly suggestible results.

As well, in most studies, concomitant drug changes were allowed, but were usually not reported; therefore, the effect of stimulation cannot be accurately measured. Finally, stimulation paradigms were frequently chosen not on a pathophysiological basis, but rather on convenience and/or chance: e.g., the 50 Hz and pulse in STN stimulus is based on previously chosen paradigms for PD treatment. There are usually four or five different stimulation variables that are freely combined, resulting in hundreds of different combinations. Further investigations are necessary to solve these limitations in this promissory area.

## Future Directions

The scope of DBS is increasing rapidly in parallel with the understanding of brain circuitry dysfunction in many pathologies (183). Investigators are only just beginning to realize the full potential of this growing field (184). The future of DBS depends on technological advances in the area: the focus should be the improvement of clinical knowledge as well as improved practicality (smaller size devices, increased battery life, greater tolerance and safety profiles, improved software). Further understanding of the mechanisms underlying cerebral circuits in epilepsy and its comorbidities is required, in order to further define specific criteria and predictors in the selection of patients who could benefit from DBS. This will be only possible investing resources in basic research, essential as it forms the foundation upon which translational research and clinical trials are built on. The poor results of DBS treatment in some patients as reported in the literature, may be mitigated in the future with improvement in patient selection, better target identification, and the development of more effective stimulation paradigms, such as closed-loop stimulation (184).

The identification of optimal targets for specific subgroups of patients, including patients with comorbidities, especially those with psychiatric disorders, is crucial for further development (185). Basic science studies have been fundamental in advancing the understanding of the complexity of alterations in neuronal networks and continue to provide valuable information for researchers and clinicians, allowing for further clinical developments, particularly in the identification of these practical targets. Modern methodologies such as EEG-fMRI studies are allowing for the delineation of epileptogenic neuronal networks, which is increasingly accepted over the classic concept of the epileptogenic zone (186). Analysis of epileptic neuronal networks will help guide treatments in a more precise way, hopefully resulting in more efficacious treatments.

Advances in surgical procedures may include the implementation of new electrodes to treat a single symptom synergistically or multiple symptoms at once, which would additionally allow for the establishment of different stimulation parameters for each electrode contact.

Finally, optogenetics rests on the use of genetically-encoded, light sensitive proteins, such as opsins, to modulate neuronal activity, intracellular signaling pathways, or gene expression with spatial, directional, temporal, and cell-type specificity (187). Epilepsy is one the disorders that has been widely explored in this regard. The ability to inhibit or activate different neurons with high specificity and resolution has turned it into an exciting research tool and a possible therapeutic intervention targeting neurons with abnormal activity (188). Studies in several animal models have shown a reduction in seizure activity both electrographically and clinically (189–191). Even so, there are still several limitations that must be overcome prior to its application in humans, particularly the ~1000-fold difference in brain volume between the rodent models and the much larger human neuronal circuits that makes optogenetic control difficult.

## Conclusion

DBS is one of the most remarkable interventions in the history of functional neurosurgery. Whether it will significantly improve the outcomes of DRE patients remains to be seen. DBS tends to have better results in patients with generalized epilepsy, although it has been used with success in some patients with focal-onset seizures. The best DBS targets for each epileptic syndrome, as well as the optimal combination of stimulation variables for each target remains speculative, however ATN stimulation has been performed in the highest number of patients and with the most rigorous study protocol allowing it to be recommended over the other targets. Results in hippocampal and frontal stimulation are suboptimal and should be reserved only for patients in whom resective procedures are contraindicated. Surgery should be recommended before potential hippocampal or frontal stimulation is considered. Targets, such as the CN and cerebellar nuclei, need more exploration. There is a need for large, well-designed randomized control trials to validate and optimize the efficacy and safety of DBS.

## Author Contributions

NZ and FS conceived and designed the analysis. NZ and LL initiated the collaborative project, collected the data, performed the analysis and wrote the paper. JO-H and LL monitored data collection, cleaned the data, and drafted the paper. AC and JT-Z revised the paper.

### Conflict of Interest Statement

JT-Z receives grants from the University of Saskatchewan, the Royal University Hospital Foundation in Saskatoon, Saskatchewan, and the Saskatchewan Health Research Foundation. AC receives grants from the Royal University Hospital Foundation in Saskatoon, Saskatchewan. The remaining authors declare that the research was conducted in the absence of any commercial or financial relationships that could be construed as a potential conflict of interest.
